# New Diagnosis of Non-compaction Cardiomyopathy in a 43-Year-Old Man Presenting with Syncope

**DOI:** 10.7759/cureus.5107

**Published:** 2019-07-09

**Authors:** Ahmad Musmar, Muhammad B Hammami, Saleh Alzaraq, Reem Aboushaar, Eli Levine

**Affiliations:** 1 Internal Medicine, Florida Atlantic University, Boca Raton, USA; 2 Medicine, Artisans of Medicine NYC, New York, USA; 3 Medicine, Florida Atlantic University, Boca Raton, USA; 4 Cardiology, Boca Raton Regional Hospital, Boca Raton, USA

**Keywords:** ncm, left ventricular non-compaction, syncope, cardiomyopathy, heart failure, cardiac mri, tte, non-compaction cardiomyopathy

## Abstract

Non-compaction cardiomyopathy (NCM) is rare congenital cardiomyopathy characterized on cardiac imaging by a two-layered ventricular wall with prominent trabeculations and intertrabecular recesses.

This case highlights a patient in his fifth decade who presented from an outpatient setting for abnormal findings found on a transthoracic echocardiogram for syncopal workup. Cardiac MRI was consistent with non-compaction cardiomyopathy. A loop recorder then inserted, and he was placed on guideline-directed therapy for heart failure with reduced ejection fraction (HFrEF) and discharged with life vest since left ventricular ejection fraction (LVEF) > 35%.

There are many areas of controversies in NCM, such as prevalence, diagnostic criteria, clinical features, prognosis, and management strategy. We will discuss the etiology, diagnostic criteria, and management.

Physicians should be aware of NCM diagnosis when a patient presents with heart failure and structural heart changes on imaging despite the age. Cardiac magnetic resonance imaging (CMRI) is the best diagnostic modality. Patients should be recognized and started on proper management to prevent complications.

## Introduction

Non-compaction cardiomyopathy (NCM) is a rare congenital cardiomyopathy which was previously called the spongy myocardium and hypertrabeculation syndrome [1,2]. It is characterized on cardiac imaging by a two-layered ventricular wall with prominent trabeculations and intertrabecular recesses that communicate with the ventricular cavity but not with the coronary circulation. The etiology is thought to be from the arrest of trabecular compaction during embryogenesis [1].

Initial presentation is variable and may occur at any age. The patient may be asymptomatic and diagnosed during a family screening or present with one of NCM complications: heart failure, arrhythmias and systemic embolic events [1].

This case highlights a patient in his fifth decade, presenting with syncope.

## Case presentation

A 43-year-old male with past medical history of NASH with prior episode of encephalopathy and hyperlipidemia, presented from an outpatient setting for abnormal findings found on a transthoracic echocardiogram for syncopal workup.

**Figure 1:**
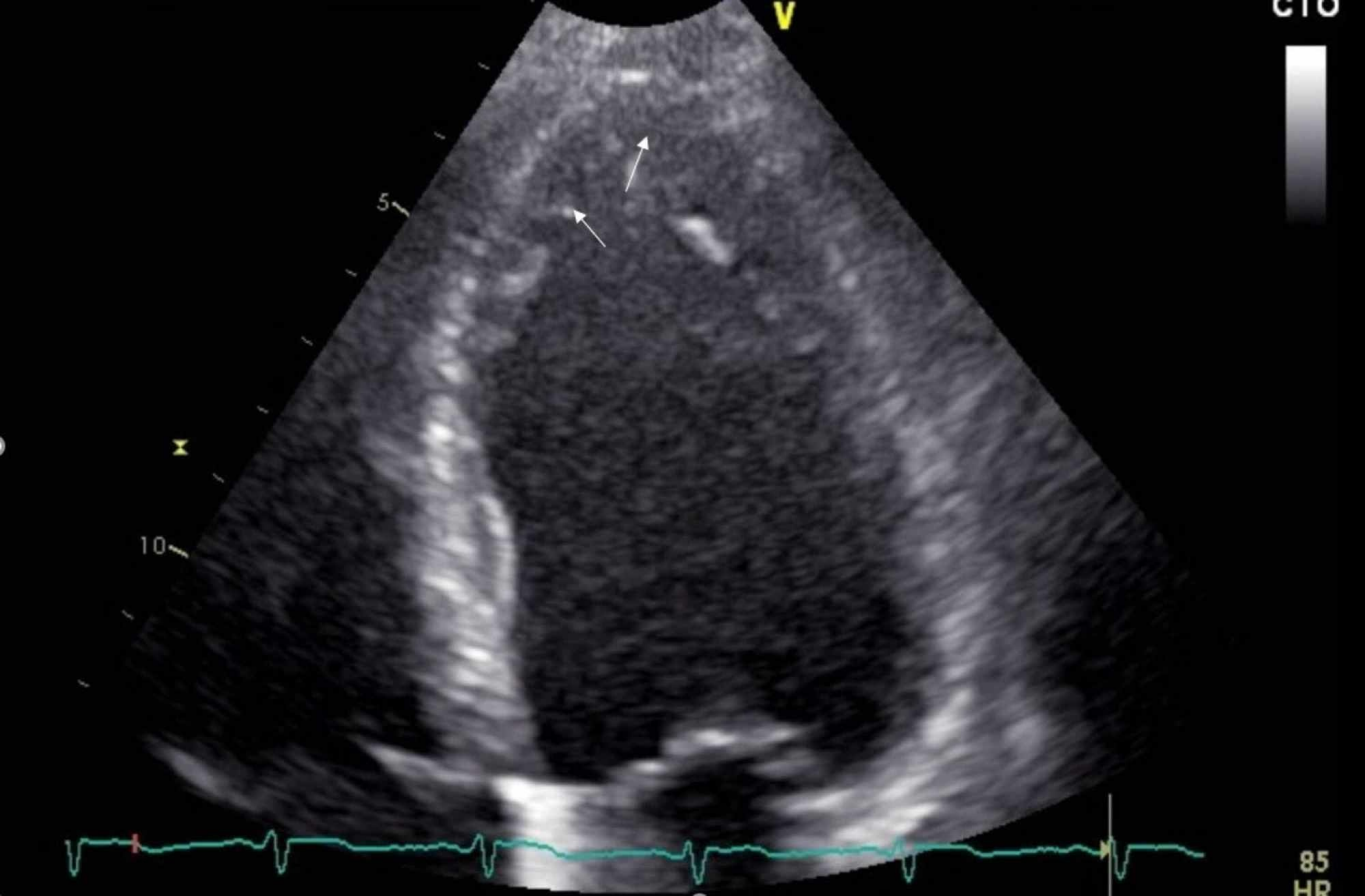
TTE image showing trabeculations (arrows) TTE: Transthoracic echocardiogram

Transthoracic echocardiogram was notable for a left ventricular ejection fraction of 40% with global hypokinesis, asymmetrical left ventricular hypertrophy, and dilated IVC dynamics (Figure 1).

The patient was admitted for syncope; two days prior to his admission he was found on the floor by his wife. No evidence of seizure was found on workup.

Upon arrival to the emergency department, he was back to his neurological baseline. He disclosed recent fatigue and decrease in exercise tolerance. He denied pre-syncopal chest pain, palpitations or shortness of breath. There was no family history of sudden cardiac death or other cardiovascular disease.

Physical exam was unremarkable besides orthostatic vital signs. Electrocardiogram showed normal sinus rhythm without any ischemic changes. Chest radiograph was unremarkable. Neuroimaging showed no intracranial pathology. CBC, CMP, and cardiac biomarkers were unremarkable. Electroencephalogram showed no abnormal tracing. CT coronary angiogram showed minimal calcification of the left main coronary artery without any significant stenosis.

**Figure 2:**
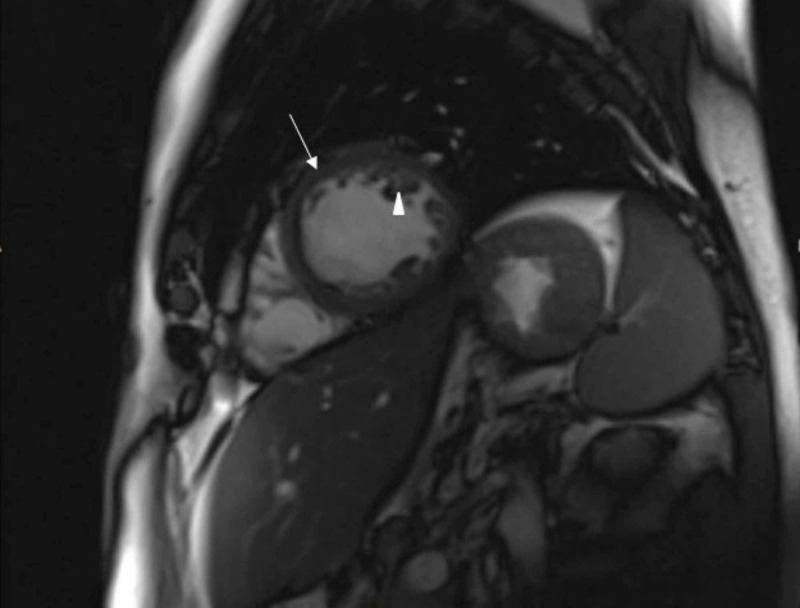
Cardiac MRI showing trabeculations (arrow head), and compacted layer (arrow)

Cardiac MRI showed decreased left ventricular ejection fraction, and global hypokinesis. Abnormal trabeculation visualized in the left ventricular apex and the lateral wall (Figure 2), with noncompacted region measuring 11 mm and compacted region measuring 4.4 mm. The noncompacted to compacted ratio was 2.5. There were no focal regions of hypertrophy within the left ventricle. There were no subendocardial enhancements.

At that point the patient met the criteria of noncompaction cardiomyopathy. The syncope is most likely secondary to an arrhythmogenic ventricular event. A loop recorder was then inserted, he was placed on guideline-directed therapy for heart failure with reduced ejection fraction (HFrEF) and discharged with life vest since left ventricular ejection fraction (LVEF) > 35%.

## Discussion

Despite being a rare entity, NCM is being diagnosed with increasing frequency as physicians are more aware of the disorder, and diagnostic modalities continue to advance [3]. The etiology of NCM is believed to be secondary to the arrest of the final stage of myocardial morphogenesis. NCM has variable array of presentations and complications include: heart failure, ventricular arrhythmias, sudden cardiac death, and thromboembolic events.

There are many areas of controversies, such as: prevalence, diagnostic criteria, clinical features, prognosis and management strategies. Imaging criteria vary and remain questionable when distinguishing NCM from normal patterns. It is still debatable whether noncompaction is a separate cardiomyopathy entity or a trait shared by different cardiomyopathies [1].

The perfect diagnostic tool, which should act as a reproducible genetic marker, is still lacking. However, echocardiogram was the diagnostic study of choice; now it is cardiac MRI with its high resolution helping to differentiate non-compacted and compacted myocardium. It also allows for the imaging of the apical wall [4]. A study by Petersen et al. using cardiovascular magnetic resonance (CMR) found that pathological non-compaction had a non-compaction to compaction ratio >2.3 in end-diastole and that the specificity and negative predictive values were both 99% [5].

The Chin et al. criteria and Jenni et al. criteria are the commonly used echocardiographic criteria [1]. Chin recommended the assessment of the ratio of X/Y dimensions obtained in diastole - where X is the distance from the epicardial surface to the trough of the trabecular recesses, and Y is the distance from the epicardial surface to the peak of the trabeculation. An X/Y ratio of up to 0.5 would be required to make the diagnosis [1].

Jenni et al. criteria take into consideration both end-diastolic and end-systolic myocardial layer thickness. NCM is diagnosed when the non-compacted/compacted ratio is more than 2.0. Prominent trabeculations must present [6].

NCM management is the treatment of its main complications - HFrEF, embolic events, and arrhythmias. Treatment is started with standard guideline-directed therapy for HFrEF with ACE-inhibitors and B blockers. Women with NCM should be cautioned about future pregnancies to mitigate complications [7]. In addition, screening of first-degree relatives with an echocardiogram is warranted due to the familial association of NCM [8].

## Conclusions

Physicians should consider NCM diagnosis when patients present with heart failure symptoms, and evidence of structural abnormalities on echocardiogram at any age. Despite several imaging diagnostic criteria using echocardiogram and cardiac MRI, the latter is the preferred diagnostic modality. NCM has serious complications as ventricular arrhythmias and thromboembolic events that can be mitigated with early diagnosis and management of heart failure.
